# *QuickStats*: Percentage* of Adults Aged ≥45 Years Who Found It Very Difficult or Were Unable to Go Shopping or Attend Movies or Sporting Events,^†^ by Degree of Hearing Difficulty^§^ and Sex — National Health Interview Survey,^¶^ United States, 2014–2015

**DOI:** 10.15585/mmwr.mm6605a7

**Published:** 2017-02-10

**Figure:**
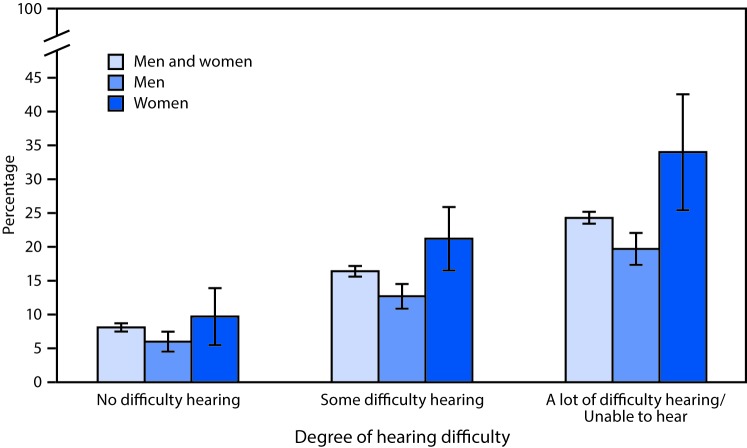
During 2014–2015, adults aged ≥45 years were more likely to find it difficult or be unable to go shopping or go to movies or sporting events as hearing difficulties increased (even with the use of a hearing aid), from 8.1% among those with no difficulty hearing to 16.4% among those with some difficulty hearing, and to 24.3% among those with a lot of difficulty hearing or who were unable to hear. This relationship was found for both men and women. Women were more likely than men to report limitations in these activities at each level of hearing difficulty.

**Source:** National Health Interview Survey, 2014–2015. https://www.cdc.gov/nchs/nhis/index.htm.

